# The Effects of Treatment in Psychotic Disorders—Changes in BDNF Levels and Clinical Outcomes: Systematic Review

**DOI:** 10.3390/ijerph20032111

**Published:** 2023-01-24

**Authors:** Anna Mosiołek, Jadwiga Mosiołek

**Affiliations:** 1 Department of Psychiatry, Faculty of Health Sciences, Medical University of Warsaw, Żwirki i Wigury 61 Street, 02-091 Warszawa, Poland; 2 John Paul II Western Hospital in Grodzisk Mazowiecki, Daleka 11 Street, 05-825 Grodzisk Mazowiecki, Poland

**Keywords:** brain-derived neurotrophic factor/BDNF, psychotic disorders, schizophrenia

## Abstract

Psychotic disorders are associated with significant impairment in functioning, and their treatment remains a great therapeutic challenge. Patients are at a higher risk of suicide and premature mortality. Biomarkers, such as brain-derived neurotrophic factor (BDNF), play a vital role in neurotransmission and neurodevelopment. Decreased levels of BDNF alter neuronal signaling and cause the appearance of symptoms such as the impairment of working memory. A literature search was performed using the PubMed data base. Following the inclusion and exclusion criteria, 24 original articles were selected. We collected available data showcasing the influence of antipsychotic and non-pharmacological treatments, in patients suffering from psychotic disorders, on clinical conditions and BDNF levels in serum or plasma. In this review, we outline emerging data regarding the influence of different antipsychotic drugs and non-pharmacological treatment methods on BDNF and discuss their role as predictors of treatment outcome. Most studies conducted with antipsychotics saw an increase in BDNF levels; however, no positive correlation between change in BDNF and PANSS scores was observed. Studies based on non-pharmacological methods varied based on the treatment applied. Therefore, it is difficult to draw definite conclusions.

## 1. Introduction

### 1.1. Psychotic Disorders

Psychotic disorders are characterized by altered brain function, resulting in significant impairments in reality testing. It is manifested by one or more of the following symptoms: disorganized thinking (switching from one topic to another), being grossly disorganized, or abnormal motor behavior, including catatonia. Delusions are defined as unchangeable beliefs that cannot be amended despite evidence proving them incorrect. Hallucinations are false sensory perceptions in the absence of external stimulus, with the most common example being hearing voices. Negative symptoms include alogia, apathy, anhedonia, affective flattening, and avolition [1,2].

The DSM-5 categorizes psychotic disorders as “Schizophrenia and other psychotic disorders” including substance-induced psychotic disorders, as well as psychotic disorder caused by other medical conditions. On the other hand, ICD-11 “Schizophrenia spectrum and other primary psychotic disorders” distinguishes a group of primary psychotic disorders, including schizophrenia, schizoaffective disorder, acute and transient psychotic disorder (ATPD), schizotypal disorder, delusional disorder, other primary psychotic disorders, and unspecified primary psychotic disorders. Nonprimary psychotic disorders, such as psychotic disorders caused by other medical conditions and psychotic disorders due to substance use or withdrawal, are categorized separately [1,2,3].

Psychotic disorders have a lifetime prevalence of approximately 3% [4,5]. Among them, schizophrenia has the highest lifetime prevalence of 0.87–1.25% [4,5]. The disorders are associated with a significant impairment of functioning and high socioeconomic costs directly related to long-term treatment, as well as due to absence from work. Furthermore, they are associated with a high risk of suicide and premature mortality [5]. Studies show that the risk of suicide during the first year of schizophrenia is 12 times higher than among the general population [6]. Up to 5% of patients will successfully commit suicide, most commonly during their first episode of psychosis [6,7]. Psychotic disorders are constantly a huge therapeutic challenge, which is why it is so important to discover new methods of early detection and treatment strategies that allow for the best possible patient care.

### 1.2. Brain-Derived Neurotrophic Factor (BDNF)

Brain-derived neurotrophic factor (BDNF) is a protein in the neurothrophin family that plays an important role in neuronal survival, growth, and plasticity. BDNF is widely expressed in the central nervous system (CNS) [8], with highest concentration levels found in the hippocampus, followed by the cerebral cortex [9]. Neurons secrete BDNF as a precursor, which is, later on, proteolytically processed into its more active form in extracellular space [10]. BDNF works through the protein tyrosine kinase receptor (TrkB). Increases in TrkB mRNA transcription and TkrB receptor endocytosis, as well as translocation are linked with neuronal activity [11].

Brain-derived neurotrophic factor is essential in the development of many areas of the nervous system [12]. It holds an important role in axonal guidance, a process required for the proper formation of neuronal connections during the development [13]. Moreover, studies conducted on animals associate the lack of neurotrophines with decreased synaptic connectivity [14]. Disruption in the process of creating neuronal pathways may contribute to the impairment of cognitive function [15].

Changes in brain-derived neurotrophic factor levels can occur in both healthy subjects, during the process of natural aging, and in those suffering from pathological conditions. Due to high concentrations of BDNF in the hippocampal area, it is more exposed to pathology related to change in its levels [16].

### 1.3. The Role of Brain-Derived Neurotropic Factor (BDNF) in Psychotic Disorders

BDNF plays a vital role in development and activity of neurotransmission, which is consistent with animal studies showcasing BDNF involvement in psychotic disorders [17,18]. Schizophrenia is the most prevalent psychotic disorder therefore studies showcasing the role of BDNF in this particular group of disorders are mostly focused on it. Neurodevelopmental model of schizophrenia suggest that reduced levels of BDNF alter neurotransmission through decreased synaptic connectivity [19,20] resulting in the appearance of symptoms. Severity of neuronal dysfunction might be a reflection of BDNF dysfunction [21]. Significant decrease in mRNA BDNF can be found in prefrontal cortex of patients suffering from schizophrenia [22,23,24]. This area of the brain is known to be involved with working memory [22]. It is consistent with present in schizophrenia deficits of all subsystems of working memory. They are observed early on and are associated with reduction in social skills and learning capacity [25].

There are few studies focusing on psychotic disorders apart from schizophrenia. It may be due to the lower prevalence of other disorders and the difficulty in finding a study-appropriate patient group. Due to this, our study focuses mainly on the role of BDNF in schizophrenia; however, we decided to include our research on other psychotic disorders. It can showcase how understudied this group of psychotic disorders, with exclusion of schizophrenia, is in the BDNF area.

## 2. Materials and Methods

### 2.1. Search Strategy

Between 23 October and 4 December of 2022, the Pub Med electronic database was systematically searched by two independent researchers. The studies included in the present research were published between 2007 and 2022. Clinical trials and randomized control trials comparing pre- and post-antipsychotic, non-pharmacological treatments serum, or plasma BDNF levels in patients suffering from psychotic disorders were considered. The search terms included: “brain derived neurotrophic factor” or BDNF, “psychotic disorder”, “schizophrenia”, “schizoaffective disorder”, “delusional disorder”, and “schizotypal disorder”.

### 2.2. Study Selection Strategy

Studies were selected for inclusion by two independent researchers, and all discrepancies were resolved by discussion. The titles and abstracts were screened, and potentially relevant studies were reviewed in full. The authors included eligible studies examining plasma or serum BDNF levels in patients pre- and post-antipsychotic and non-pharmacological treatments. Furthermore, each included study should state the period of the treatment and at what points in time BDNF measurements were taken. Abstracts, case studies, family-based designs, population-based studies on healthy subjects, systematic reviews, meta-analyses, and duplicate cohorts were excluded.

### 2.3. Data Extraction and Outcome Measures

**Figure 1:**
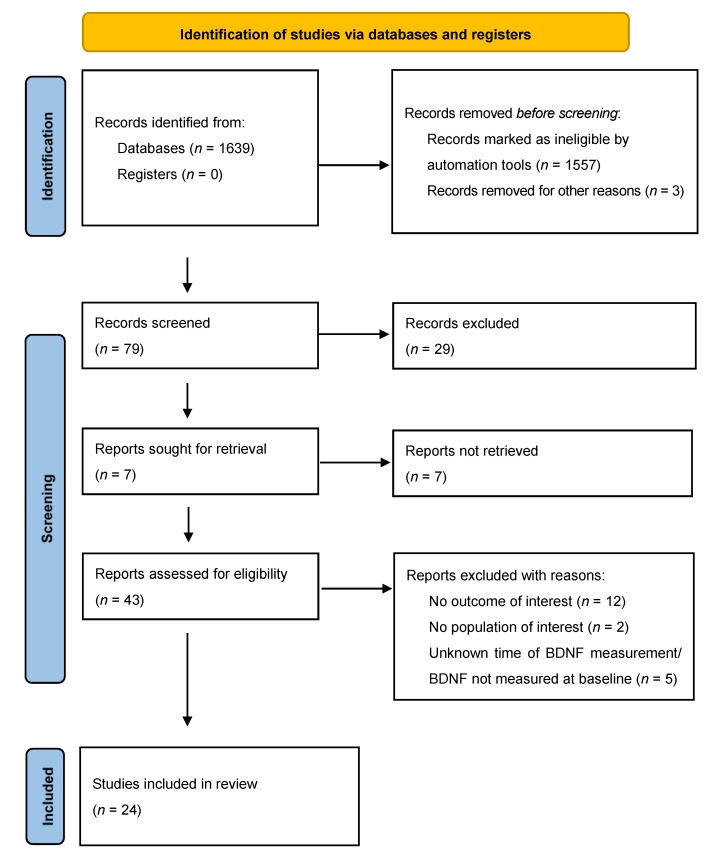
The PRISMA 2020 statement. [26].

There were two authors who independently extracted data to avoid extraction errors. The research was carried out with the use of the PRISMA flow diagram (Figure 1). The following parameters were extracted from each eligible article: first author, publication year, language of the full text, diagnostic system, number of subjects, treatment used, and measuring sample (plasma or serum) BDNF levels. The primary outcome was serum or plasma BDNF levels from baseline to post-treatment. If one study reported BDNF concentrations for more than one time point within our pre-defined periods, we considered the data recorded at baseline and the last time point within the range for the overall effect analysis.

### 2.4. Selection Criteria

Selection criteria for the present systematic review were as follows: clinical diagnosis of psychotic disorder (DSM-5 and ICD-10); duration of at least 6 weeks in studies based on antipsychotic medication and 4 weeks for studies based on non-pharmacological treatment; assessment of plasma or serum BDNF levels, at least in two points in time, at baseline and at the end of treatment; medication-based studies with any of the following drugs: risperidone, paliperidone, olanzapine, lurasidone, memantine, ziprasidone, aripiprazole; non-pharmacological studies, including treatment methods such as: repetitive transcranial magnetic stimulation sessions, electroconvulsive therapy, electroacupuncture, cognitive remediation treatment, rehabilitation, biofeedback, computerized auditory training, sarcosine, n3 polyunsaturated fatty acids, exercise sessions, yoga, and omega-3 fatty acids. The search terms included: psychotic disorders (defined by DSM5 and ICD-10) and brain derived neurotrophic factor (BDNF). Out of 1639 citations obtained in our initial search 24 articles met the inclusion criteria.

## 3. Results

### 3.1. Influence of Pharmacological Treatment on BDNF

The influence of antipsychotic medication on BDNF levels is still not as well-known as the influence of antidepressants on depression. There are a few studies on the topic, and most of them are conducted with atypical antipsychotic medication [27,28,29,30,31,32,33,34]. As BDNF levels differ in serum and plasma, we decided to observe its behavior separately for both. The results are summarized in Table 1.

#### 3.1.1. Serum BDNF

Out of four studies included in our review, three were 12 weeks long [27,28,30], and one lasted for 6 weeks [29]. Only atypical antipsychotics (risperidone, paliperidone, lurasidone, paliperidone, olanzapine, clozapine) were used. Memantine was used as an add-on treatment to clozapine in one of the studies; however, no differences in mean serum BDNF levels before and after memantine or placebo treatments were observed. It is worth mentioning that this study was conducted on a relatively small sample (*N* = 10), all participants were on clozapine before entering the trial, and the increase in BDNF levels might have occurred before the first BDNF measurement [27].

There were two studies conducted on similar patient groups (*N* = 45/*N* = 47 [28] and *N* = 47/*N* = 47 [28]) that observed an increase in serum BDNF levels in all medication groups (lurasidone, olanzapine, paliperidone, risperidone). The rise in BDNF levels in the olanzapine group was found to be significantly higher than in the lurasidone group; however, neither showed any correlation between the rises in BDNF and PANSS scores [29]. The other study found a significant increase in BDNF levels in both treatment groups and an improvement in cognitive functioning. Negative correlation between the reduction rate of the PANSS score and the increase in serum BDNF level was noted after the treatment with paliperidone but not with risperidone [28].

#### 3.1.2. Plasma BDNF

Six studies focusing on BDNF levels in plasma took between 6 and 12 weeks of therapy with one of the atypical antipsychotics: risperidone, olanzapine, ziprasidone, aripiprazole, or paliperidone [30,31,32,33,34]. There were two studies that did not observe alteration in BDNF levels following treatment [31,34]. There were three studies that noted a significant increase in BDNF levels [21,32,33]. Double blinded trial, comparing response to risperidone + placebo vs. risperidone + dextromethorphan, did not find a significant correlation between BDNF levels and PANSS scores; however, the plasma BDNF levels were significantly negatively correlated with PANSS negative syndrome scores [21]. A study using aripiprazole as a method of treatment reported a negative correlation between the duration of psychosis and plasma BDNF levels. There was no correlation between plasma BDNF levels and the dose of aripiprazole [33].

### 3.2. Influence of Non-Pharmacological Treatment on BDNF

Non-pharmacological treatment methods have a great influence on the course of overall therapy. In this section, we would like to investigate the role of BDNF in different types of non-pharmacological treatments used in psychotic disorders. We collected data on a wide variety of available treatment methods, such as repetitive transcranial magnetic stimulation (rTMS), electroconvulsive therapy (ECT), electroacupuncture, cognitive remediation treatment (CRT), rehabilitation, and biofeedback, among others, as well as their influence on BDNF, and we gathered them in Table 2. We present in-depth findings in text below.

#### 3.2.1. Serum BDNF

The study on schizophrenia patients undertaking rTMS sessions applied to the supplementary motor area with serum BDNF levels that were measured at three points in time, alongside clinical assessment, by a psychiatrist and showed no significant findings [35]. Similarly, a study from 2017, in which rTMS was applied to the right inferior parietal lobule of schizophrenia patients, did not observe significant findings. It is necessary to note that the groups of patients in both studies were small (*N* = 6 and *N* = 8 respectively); therefore, it is not possible to draw clear conclusions [36].

There were 30 schizophrenia patients who underwent 4 weeks (3 sessions a week) of electroacupuncture. Serum BDNF levels were measured at baseline, as well as after the end of treatment and clinical assessment was conducted, using the Positive and Negative Syndrome Scale (PANSS). Memory Scale (WMS) and Wisconsin Card Sorting Test (WCST) were used to do the cognitive assessment. No significant differences were found in the mean serum BDNF levels between baseline and at the end of treatment; however, a positive association was found between the increase in serum BDNF and memory improvement but not with other cognitive functions. The authors of the study indicate that EA positively associates cognitive benefits with improved BDNF levels [38].

Other studies focusing on serum BDNF levels included treatment methods such as: cognitive remediation treatment (CRT), aerobic exercise, computerized auditory training, sarcosine, standard rehabilitation, and biofeedback [39,40,41,42,44,45]. CRT is often used in schizophrenia therapy, and the study assessing CRT and serum BDNF levels showed that both quality of life and cognition were improved; however, no association with BDNF levels was found [39]. Similarly, sarcosine-taking patients noted improvement in negative symptoms and PANSS score, but there was no correlation with BDNF levels [41]. On the contrary, rehabilitation and biofeedback improved the PANSS score, attention, and concentration alongside the increase in BDNF [40]. A study with the use of computerized auditory training also showed a significant increase in serum BDNF levels and correlated with improved quality of life [42]. Studies concerning the effects of aerobic exercise on patients with psychotic disorders are inconsistent [44,45], with one study (only schizophrenia patient group) observing an increase in BDNF levels and improvement in neurocognitive functioning [45] and the other (schizophrenia and other psychotic disorders patient group) showcasing no significant findings [44].

#### 3.2.2. Plasma BDNF

Studies measuring plasma BDNF levels included treatment methods such as electroconvulsive therapy (ECT), n-3 PUFA, Omega-3 fatty acids, hatha yoga, exercise, and cognitive behavioral therapy (CBT) [37,43,46,47,48,49]. Cognitive Behavioral Therapy (CBT) as an adjunctive to treatment-as-usual, in a group of eighty-six patients with first-episode psychosis, observed an increase in plasma BDNF levels associated with a decrease in general psychotic symptoms [47]. Similarly, usage of omega-3 fatty acids was also associated with an increase in BDNF levels. Moreover, schizophrenia patients with metabolic syndrome noted improvement in cognitive functioning. Although only conducted on a small sample group, aerobic exercise showed a protective effect on BDNF, which increased in the exercise intervention group but decreased in the control group (treatment as usual) [48]. In contrast, hatha yoga did not show positive outcomes in patients suffering from schizophrenia spectrum disorders [46]. Interestingly, 26 weeks of daily intake of n-3 PUFFA increased plasma BDNF levels; however, it showed a significant negative correlation between change in BDNF (at baseline and at the end of the study) and the score of the Calgary Depression Scale for Schizophrenia (CDSS) [43].

## 4. Discussion

In this study, we show the impact of various forms of therapy on BDNF levels and their association with clinical response in patients suffering from psychotic disorders. We observed that response to antipsychotic medication varied. Serum BDNF levels in most studies increased; however, there was no significant correlation with clinical response to treatment [28,29,30], despite improved cognitive functioning [28]. Additionally, negative correlation between the reduction rate of the PANSS score and the increase in serum BDNF level was observed in patients taking paliperidone [28]. Similar results were observed with plasma BDNF levels. The studies that reported an increase in BDNF levels did not show significant correlation with PANSS scores and clinical response [21,30,32].

Different forms of non-pharmacological treatment were considered. Studies on rTMS did not report significant findings; however, we must consider the limitations of both studies, as they were conducted on a small sample [35,36]. Similarly, hatha yoga therapy did not show any significant results [46]. Cognitive remediation treatment and CRT with add-on aerobic exercise improved cognitive functioning and quality of life without significant changes in BDNF levels [39,44]. On the contrary, standalone aerobic exercise increased plasma [49] and serum [45] BDNF levels; however, there was no significant correlation with clinical symptoms [45,49]. Electroacupuncture showed a significant positive correlation with memory improvement; however, it is hard to make clear conclusions from just one study [38].

The limitations of this study include low repeatability of treatment methods applied in presented studies, which makes it difficult to draw clear conclusions. It is important to conduct follow up studies to obtain more accurate results, especially with the use of treatment methods that showed potential positive results in patients. It also has to be noted that most studies focused on schizophrenia, and only a few included other types of psychotic disorders. Moreover, in most cases, patients were previously taking antipsychotic medication before the beginning of the study, which can affect BDNF levels. The authors believe that more studies should focus on other types of psychotic disorders than schizophrenia, especially on the first episode, as well as drug-naïve patients, as previous medication may influence BDNF levels.

## 5. Conclusions

In this review, we considered a variety of antipsychotic therapies, their influence on serum or plasma BDNF levels, and their correlation with clinical response. Our findings for both pharmacological and non-pharmacological treatments show that an increase in BDNF levels does not correlate with clinical response to treatment. Electroacupuncture showed significant positive correlation with memory improvement; however, more studies on a bigger sample are needed to make clear conclusions. Limitations of our review are that treatment methods varied, and it is difficult to draw conclusions based of such a diverse group.

## Figures and Tables

**Table 1:** The influence of antipsychotic treatment on BDNF.

Study	Year of Publication	Type of Psychotic Disorder	Medications	Duration of the Study	Serum/Plasma BDNF	Study Group (*N*)	Time of BDNF Levels Measurement	Main Findings
de Lucena et al. [27]	2010	schizophrenia	memantine as adjunctive therapy to clozapine	12 weeks	serum BDNF	*N* = 10	Measured at baseline and after 12 weeks of treatment.	No significant findings.
Wu et al. [28]	2018	schizophrenia	risperidone, paliperidone	12 weeks	serum BDNF	*N* = 47 (risperidone) *N* = 47 (paliperidone)	Measured at baseline and after 12 weeks of treatment.	Significant increase in BDNF levels and improvement in cognitive functioning in both treatment groups. Paliperidone group: negative correlation between the reduction rate of the PANSS score and the increase in serum BDNF level.
Jena et al. [29]	2019	schizophrenia	olanzapine, lurasidone	6 weeks	serum BDNF	*N* = 47 (olanzapine) *N* = 45 (lurasidone)	Measured at baseline and after 6 weeks of treatment.	There was an increase in BDNF levels in both treatment groups. No significant correlation between PANSS score and serum BDNF.
Wu et al. [30]	2022	schizophrenia	risperidone	12 weeks	plasma BDNF serum BDNF	*N* = 183	Measured at baseline and after 12 weeks of treatment.	Slight increase in BDNF levels in both plasma and serum.
Hori et al. [31]	2007	schizophrenia	olanzapine	8 weeks	plasma BDNF	*N* = 32	Measured at baseline and after 8 weeks of treatment.	Olanzapine did not alter BDNF levels.
Silver et al. [32]	2011	schizophrenia	risperidone: *n* = 3, olanzapine: *n* = 1, ziprasidone: *n* = 3, typical antipsychotics *n* = 7 + add on Fluvoxamine	6 weeks	plasma BDNF	*N* = 14	Measured at baseline (before addition of fluvoxamine) and after 1, 3 and 6 weeks of treatment.	Plasma BDNF levels concentrations were increased after treatment.
Chen et al. [21]	2012	schizophrenia	risperidone + placebo/Dextromethorphan	11 weeks	plasma BDNF	*N* = 95	Measured at baseline and after 2, 4, 8, and 11 weeks of treatment.	Increase in BDNF in both groups. There was no significant correlation of plasma BDNF levels with the PANSS scores.
Yoshimura et al. [33]	2012	schizophrenia	aripiprazole	8 weeks	plasma BDNF	*N* = 50	Measured at baseline and after 8 weeks of treatment.	Significant increase in plasma BDNF levels. A negative correlation between duration of psychosis and plasma BDNF levels.
Chung et al. [34]	2017	schizophrenia	paliperidone	8 weeks	plasma BDNF	*N* = 51	Measured at baseline and after 8 weeks of treatment.	BDNF did not increase after 8 weeks of treatment.

**Table 2:** The influence of non-pharmacological treatment in psychotic disorders on BDNF.

Study	Year of Publication	Type of Psychotic Disorder	Applied Therapy	Duration of the Study	Serum/Plasma BDNF	Study Group (*N*)	Time of BDNF Levels Measurement	Main Findings
Mendes-Filho et al. [35]	2016	schizophrenia schizoaffective disorder	Repetitive transcranial magnetic stimulation (rTMS) applied to the supplementary motor area (1 Hz, 20 min, 20 sessions)	4 weeks	serum BDNF	*N* = 6	At baseline, at the end of treatment and 4 weeks after the treatment	No significant findings.
Tikka et al. [36]	2017	schizophrenia	rTMS applied to right inferior parietal lobule	2 weeks	serum BDNF	*N* = 8	At baseline and post-rTMS sessions	No significant findings.
Ivanov et al. [37]	2019	treatment-resistant paranoid schizophrenia	Electroconvulsive therapy (ECT) + haloperidol kolpiks, olanzapine, quetiapine, aripiprazole, paliperidone, clozapine	4 weeks	plasma BDNF	*N* = 66	At baseline and after the end of ECT	BDNF had an upward trend in subjects with combined electroconvulsive and antipsychotic treatment.
Sun et al. [38]	2016	schizophrenia	Electroacupuncture	4 weeks	serum BDNF	*N* = 30	At baseline, after treatment	Significant positive correlation between the increase in BDNF levels and memory improvement.
Penadés et al. [39]	2017	schizophrenia	Cognitive remediation treatment (CRT)	4 months	serum BDNF	*N* = 35	At baseline, after 4 weeks, at the end of treatment	Improvement in both cognitive functioning and quality of life, without significant changes in BDNF levels.
Markiewicz et al. [40]	2021	schizophrenia	Group 1—patients followed a standard rehabilitation Group 2—patients received GSR Biofeedback (galvanic skin response Biofeedback)	3 months	serum BDNF	*N*1 = 26 *N*2 = 18	At baseline, after treatment	Significant improvement of symptoms measured on the PANSS and improved attention and concentration in both groups. Group 2 noted higher statistically significant increase in BDNF levels than Group 1.
Strzelecki et al. [41]	2016	schizophrenia	Sarcosine	6 months	serum BDNF	*N* = 27	At the beginning, after 6 weeks and after 6 months	Improvement in negative symptoms, general psychopathology and total PANSS, however there was no correlations between serum BDNF (stable levels throughout the study) concentrations and PANSS scores.
Vinogradov et al. [42]	2009	schizophrenia	Computerized auditory training	10 weeks	serum BDNF	*N* = 56	At baseline, after 2 weeks and after 10 weeks	Significant increase in BDNF and cognitive gains.
Pawełczyk et al. [43]	2019	schizophrenia	2.2 g/day of n-3 PUFA (n-3 polyunsaturated fatty acids)	26 weeks	plasma BDNF	*N* = 36	At baseline and after 8 weeks and 26 weeks	A significant negative correlation was seen between the change from baseline to week 26 for plasma BDNF and the score of the Calgary Depression Scale for Schizophrenia (CDSS).
McGurk et al. [44]	2021	schizophrenia, schizoaffective disorder, schizophreniform disorder	Add on aerobic exercise program to intensive cognitive remediation program (CR + E)	10 weeks	serum BDNF	*N* = 15	At baseline, pre-and post-exercise at 5-weeks and 10-weeks	Aerobic exercise did not change the effects of cognitive remediation in patients with psychotic disorders. No significant changes in BDNF levels were noticed.
Kimhy et al. [45]	2015	schizophrenia	Aerobic exercise AE program utilizing active-play video games (Xbox 360 Kinect) and traditional AE equipment	12 weeks	serum BDNF	*N* = 13	At baseline and 12th week	Aerobic exercise enhanced neurocognitive functioning. Increase in BDNF levels were noted.
Ikai et al. [46]	2014	schizophrenia-spectrum disorders	Hatha yoga	8 weeks	plasma BDNF	*N* = 25	At baseline, after 8 weeks	No significant findings.
González-Ortega et al. [47]	2021	first-episode psychosis (FEP)	Cognitive Behavioral Therapy (CBT) as an adjunctive to treatment-as-usual (TAU)	6.5–7.5 months	plasma BDNF	*N* = 86	At baseline, post treatment	The CBT + TAU participants exhibited a greater decline in depressive, negative, and general psychotic symptoms and a greater increase in BDNF levels than controls.
Tang et al. [48]	2020	schizophrenia with metabolic syndrome (MetS)	Omega-3 fatty acids	12 weeks	plasma BDNF	*N* = 37	At baseline and 12 weeks	Omega-3 fatty acids have beneficial effects on cognitive function in patients with MetS, which is paralleled by enhanced BDNF levels.
Fisher et al. [49]	2020	first-episode psychosis (FEP)	Exercise sessions	12 weeks	plasma BDNF	*N* = 7	At baseline, after 6 and 13 weeks	Increase in BDNF levels in patients undergoing exercise sessions.

## Data Availability

The data presented in this study are openly available in PubMed at https://pubmed.ncbi.nlm.nih.gov accessed on 29 October 2021.
